# Drought Stress Causes a Reduction in the Biosynthesis of Ascorbic Acid in Soybean Plants

**DOI:** 10.3389/fpls.2017.01042

**Published:** 2017-06-15

**Authors:** Amaia Seminario, Li Song, Amaia Zulet, Henry T. Nguyen, Esther M. González, Estíbaliz Larrainzar

**Affiliations:** ^1^Department of Environmental Sciences, Universidad Pública de NavarraPamplona, Spain; ^2^Division of Plant Science and National Center for Soybean Biotechnology, University of Missouri, ColumbiaMO, United States

**Keywords:** ascorbic acid, drought stress, legumes, Smirnoff-Wheeler pathway, soybean

## Abstract

Drought provokes a number of physiological changes in plants including oxidative damage. Ascorbic acid (AsA), also known as vitamin C, is one of the most abundant water-soluble antioxidant compound present in plant tissues. However, little is known on the regulation of AsA biosynthesis under drought stress conditions. In the current work we analyze the effects of water deficit on the biosynthesis of AsA by measuring its content, *in vivo* biosynthesis and the expression level of genes in the Smirnoff-Wheeler pathway in one of the major legume crop, soybean (*Glycine max* L. Merr). Since the pathway has not been described in legumes, we first searched for the putative orthologous genes in the soybean genome. We observed a significant genetic redundancy, with multiple genes encoding each step in the pathway. Based on RNA-seq analysis, expression of the complete pathway was detected not only in leaves but also in root tissue. Putative paralogous genes presented differential expression patterns in response to drought, suggesting the existence of functional specialization mechanisms. We found a correlation between the levels of AsA and GalLDH biosynthetic rates in leaves of drought-stressed soybean plants. However, the levels of *GalLDH* transcripts did not show significant differences under water deficit conditions. Among the other known regulators of the pathway, only the expression of *VTC1* genes correlated with the observed decline in AsA in leaves.

## Introduction

Drought stress is one of the main factors limiting plant productivity in agriculture. At the molecular level, drought provokes an increase in the formation of reactive oxygen species (ROS) in plants, leading to oxidative stress and cell damage (Hsiao, 1973). In order to cope with this increased ROS production, plant cells display a complex array of both enzymatic and non-enzymatic detoxification mechanisms. The latter group includes the production of low-molecular weight compounds such as AsA (vitamin C), glutathione (GSH), carotenoids or flavonoids (Mittler, 2002; Mittler et al., 2004; Szarka et al., 2012). AsA is one of the most abundant water-soluble reducing compound present in plant tissues, serving also as an electron donor in numerous reactions (Foyer and Noctor, 2011). Synthesized in mitochondria, AsA is present in all cellular compartments at concentrations up to 21 mM (Szarka et al., 2013) and it can represent more than 10% of the soluble carbohydrate fraction in plants (Noctor and Foyer, 1998). AsA is oxidized to monodehydroascorbate and then to DHA, which can be recycled back to AsA by the AsA-GSH cycle, also known as the Foyer-Halliwell-Asada pathway (Foyer and Halliwell, 1976; Asada, 1999).

Although alternative pathways have been described, molecular genetic evidence from the model plant *Arabidopsis thaliana* indicates that the Smirnoff-Wheeler or D-mannose/L-galactose pathway is the primary route of AsA biosynthesis in plants (Wheeler et al., 1998; Conklin et al., 1999; Dowdle et al., 2007). Despite the importance of AsA in plants, knowledge on the mechanisms regulating its biosynthesis and metabolism is still limited. The steady state level of transcripts encoding several of the Smirnoff-Wheeler biosynthetic enzymes has been shown to correlate with exposure to light and AsA content (Gatzek et al., 2002; Tamaoki et al., 2003; Bartoli et al., 2006, 2009; Dowdle et al., 2007; Maruta et al., 2008; Yabuta et al., 2008; Fukunaga et al., 2010). Besides this light-dependent regulation, two genes have been identified as regulators of the pathway in *A. thaliana*: AMR1, a predicted F-box protein (Zhang et al., 2009) and VTC3, a protein kinase::protein phosphatase (Conklin et al., 2013).

Abiotic stresses are known to activate ROS-scavenging mechanisms such as superoxide dismutase, ascorbate peroxidase, and catalase enzymes (Mittler, 2002). It could be expected that drought stress would trigger an increase in the biosynthesis of a major antioxidant compound like AsA. Consequently, plants with increased AsA levels might present improved tolerance to such stresses. This is particularly relevant in the case of legume crops, main source of protein for humans and animal feed, which are highly sensitive to environmental constrains (Tuteja et al., 2012). Nevertheless, it remains unclear whether drought stress leads to an increased production of AsA. For instance, AsA levels decreased in spinach but not in soybean leaves as Ψ_w_ values dropped (Robinson and Bunce, 2000). Similarly, drought stress has been shown to cause increased AsA content in chloroplasts but a general reduction of AsA levels at the whole leaf level in several plant species (Munné-Bosch and Alegre, 2003). At the enzymatic level, AsA biosynthesis is found stimulated in mitochondria isolated from plants treated with gibberellic acid (Millar et al., 2003), a hormone inhibited during drought stress. Additionally, Bartoli et al. (2005) did not find a correlation between drought stress and AsA biosynthesis, being GalLDH activity and AsA content in wheat leaves independent on the stress tolerance of the cultivar.

The main aim of the current work is to identify the effects of drought stress on the biosynthesis of AsA in soybean plants analyzing its content, biosynthetic activity rates and the expression level of genes in the Smirnoff-Wheeler biosynthesis pathway in several plant tissues. We identified the putative orthologous genes encoding enzymes in the pathway and compared their expression levels under well-watered and drought stress conditions relative to AsA levels. Our results show that there is a significant genetic redundancy of the pathway in soybean, and to a lesser extent in *M. truncatula*, with multiple gene copies for each step in the pathway showing differential patterns of expression under drought stress conditions.

## Materials and Methods

### Plant Growth Conditions, Drought Treatments, and Water Status Characterization

*Glycine max* (L.) Merr. cv Oxumi seeds were sterilized as previously described (Labhilili et al., 1995), germinated for 3 days in the darkness at 26°C and grown in 1-L pots containing 2:1 (v/v) vermiculite:perlite under controlled conditions (24°C/18°C day/night temperature, 60/70% day/night relative humidity and 16-h photoperiod). Plants were watered three times a week with a nutrient solution (Rigaud and Puppo, 1975) supplemented with 5 mM KNO_3_. Four-week-old plants were separated into two sets: control and drought. Control plants were supplied daily with the nutrient solution to field capacity, whereas drought was achieved by withholding water/nutrients. Two hours after the start of the photoperiod, leaf and root Ψ_w_ were measured. Leaf Ψ_w_ was measured in the first fully expanded leaf using a pressure chamber (Soil Moisture Equipment, Santa Barbara, CA, United States) (Scholander et al., 1966). Root Ψ_w_ was measured using C52 sample chambers coupled to a HR-33T Dew Point microvoltmeter (Wescor). Soybean leaf Ψ_w_ was daily monitored to classify plants into control (C; leaf Ψ_w_ = -0.23 ± 0.04 MPa), VMS (leaf Ψ_w_ = -0.54 ± 0.02 MPa) and MS (leaf Ψ_w_ = -1.13 ± 0.1 MPa). Plant tissue FW was measured and aliquots were collected, immediately frozen in liquid nitrogen and stored at - 80°C for analytical determinations. The remaining tissue was employed for DW determinations after drying for 48 h at 70°C. WC was calculated using the following formula: WC = [(FW-DW)/FW] × 100.

### Determination of Total and Reduced AsA Content

Ascorbic acid content was measured by high-performance capillary electrophoresis as previously described (Davey et al., 1996). Frozen leaf, stem and root samples (∼0.2 g FW) were homogenized using a mortar and pestle under liquid nitrogen and mixed with 1.5 mL 2% (v/v) metaphosphoric acid containing 1 mM ethylenediaminetetraacetate. Samples were centrifuged (12 min, 13000 g, 4°C) and the filtered supernatant was used to determine antioxidants by capillary electrophoresis using a buffer containing 60 mM NAH_2_PO_4_, 60 mM NaCl (pH 7), and 0.0001% hexadimethrine bromide under the following conditions: -15 kV potential, 50 μm-internal diameter and 30/40.2 cm-long capillary tube with indirect UV detection at 256 nm. To obtain total AsA pools, samples were treated with dithiothreitol. DHA levels were calculated as the difference between the total AsA pool and the reduced form.

### *In Vivo* AsA Biosynthesis Assay

*In vivo* biosynthesis of AsA was assayed as previously reported (Bartoli et al., 2000). Briefly, leaf and root samples (∼0.1 g FW) were sliced and incubated for 3 h at 25°C in a buffer containing 50 mM Tris-HCl (pH 8.0) either with or without 50 mM GalL. *In vivo* AsA synthesis capacity was estimated as the difference in AsA content between time 3 h vs. time 0 in untreated samples and samples supplemented with GalL.

### Gene Identification and Phylogenetic Analysis

The putative orthologous genes implicated in Smirnoff-Wheeler pathway were identified by BLASTP (cutoff *E*-value < 1E^-10^, identity > 60%) using the following *A. thaliana* proteins: VTC1 (GDP-D-mannose pyrophosphorylase, At2g39770.1), GME (GDP-D-mannose 3′,5′-epimerase, At5g28840.1), VTC2/VTC5 (GDP-L-galactose phosphorylase, At4g26850.1 and At5g55120.1, respectively), VTC4 (L-galactose-1-P phosphatase, At3g02870.1), GalDH (L-galactose dehydrogenase, At4g33670.1), GalLDH (At3g47930.1) and VTC3 (a protein kinase, AT2G40860.1). Search was carried out against the corresponding protein sequences of the Soybean Knowledge Base genome (version Wm82.a1.v1.1) ^1^ (Joshi et al., 2014) and the *Medicago truncatula* genome (version 4.0^2^).

The predicted amino acid sequences for each step in the pathway were aligned using MAFFT^3^ using G-INS-i as iterative refinement method and BLOSUM30 as scoring matrix. Phylogenetic analyses were conducted in MEGA7 (Kumar et al., 2016) using the Maximum Likelihood method based on the JTT amino acid substitution model and 1000 bootstrap replications.

### Expression Analysis

Soybean gene expression values were obtained using RNA-seq as previously described (Song et al., 2016). Differential gene expression analysis was performed using Cuffdiff (version v2.1.1) among the different sample comparisons. For soybean, only genes with more than a twofold change and a *p*-value less than 5^∗^10^-5^ were considered as significant differentially expressed genes (Student’s *t*-test). The expression profile of the *M. truncatula* orthologs was retrieved from the Medicago Gene Expression Atlas^4^ (Benedito et al., 2008), extended with data on drought stress responses (Zhang et al., 2014). For *M. truncatula*, genes changing more than a twofold compared to control and a *p*-value less than 0.005 were considered differentially expressed (Student’s *t*-test). The average from three biology replicates was used to calculate the fold change in gene expression. Relative expression results were calculated as ratios taking expression values at day 0 as a reference.

## Results

### Physiological Characterization of Soybean Plants under Drought Stress

**FIGURE 1:**
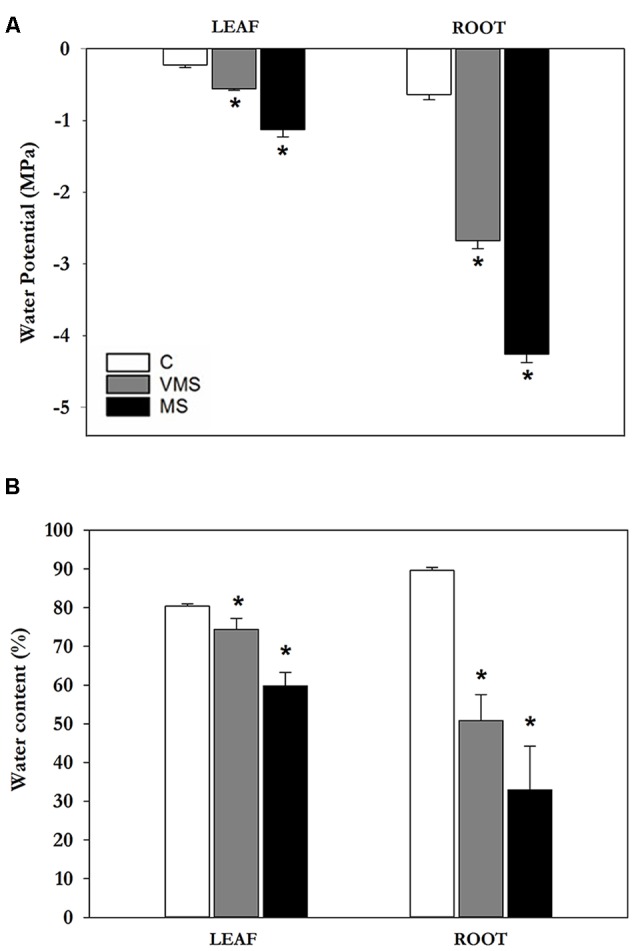
Effects of progressive drought stress on leaf and root water potentials **(A)** and WC **(B)** in soybean plants under well-watered control (C), VMS and MS conditions. Values represent the average ± SE (*n* = 4 biological replicates). An asterisk (^∗^) denotes significant differences (Student’s *t*-test, *p* ≤ 0.05) with respect to C plants.

Soybean plants were subjected to progressive drought stress by withholding water/nutrients and leaf Ψ_w_ was monitored to determine the level of stress. Plants were assigned to the following groups: well-watered plants were used as controls (C; leaf Ψ_w_ = -0.23 ± 0.04 MPa); 3 days after the onset of drought plants were classified as VMS (leaf Ψ_w_ = -0.54 ± 0.02 MPa) and 7 days after the onset of drought plants were under MS conditions (MS; leaf Ψ_w_ = -1.13 ± 0.1 MPa) (**Figure 1A**). To better characterize the plant water status, we also measured root Ψ_w_ (**Figure 1A**) and the relative WC (**Figure 1B**). A significant decline in Ψ_w_ was observed both in leaves and roots of plants subjected to progressive drought. In leaves, MS caused an almost fivefold decline in Ψ_w_ compared to C plants. In roots, Ψ_w_ values declined fourfold in VMS and sixfold in MS conditions, reaching more negative values compared to the aerial part. Leaf and root WC in C plants showed values around 80–90% throughout the study period (**Figure 1B**). Water deprivation caused a gradual decrease in WC in both organs, which was more pronounced in roots (-63% vs. -26%; **Figure 1B**). This response is consistent with the observed decline in Ψ_w_ values in the different drought treatments and provides a physiological context to the progressive drought stress imposed.

### AsA Content and *In Vivo* AsA Synthesis Decline in Leaves of Drought-Stressed Soybean Plants

**FIGURE 2:**
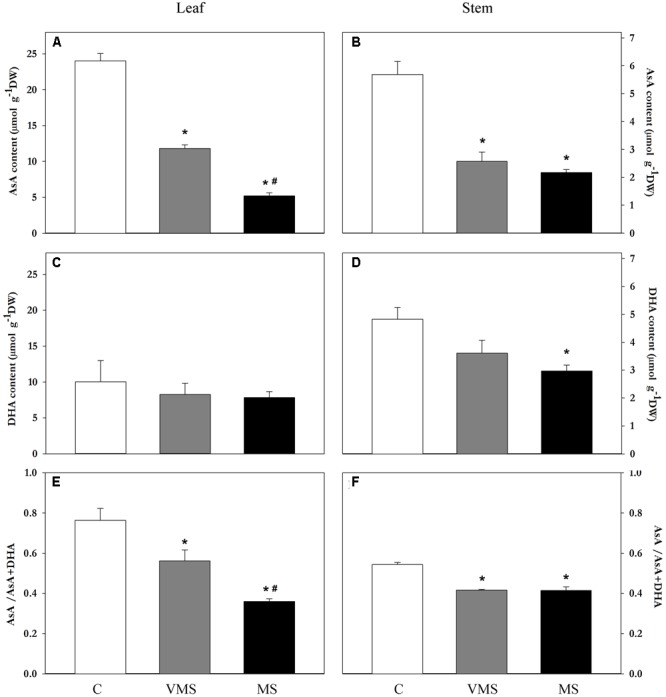
Variation on the levels of AsA, DHA, and AsA/AsA+DHA on leaf **(A,C,E)** and stem **(B,D,F)** samples of soybean plants under control (C), VMS and MS conditions. Values represent the average ± SE (*n* = 3 biological replicates). An asterisk (^∗^) denotes significant differences (Student’s *t*-test, *p* ≤ 0.05) with respect to C plants. A hash (#) denotes significant differences (Student’s *t*-test, *p* ≤ 0.05) between VMS and MS plants.

To investigate whether the levels of AsA were affected by drought, the content of AsA and DHA were monitored in leaf, root and stem samples using high-performance capillary electrophoresis. Leaves showed a higher content of both AsA and DHA compared to stems under control conditions (fourfold and twofold higher, respectively; **Figures 2A–D**), presenting also a higher reduced/total AsA ratio (0.76 vs. 0.54, respectively; **Figures 2E,F**). Drought stress caused a progressive reduction of the levels of AsA in both aerial tissues (**Figures 2A,B**), while the content of DHA was only significantly reduced in stem samples (**Figure 2D**). This suggests that the decline in AsA levels during drought is not explained by a conversion to DHA. Interestingly, the content of AsA and DHA in soybean root samples was found under the detection limit of the technique (0.75 μmol g^-1^ DW).

**FIGURE 3:**
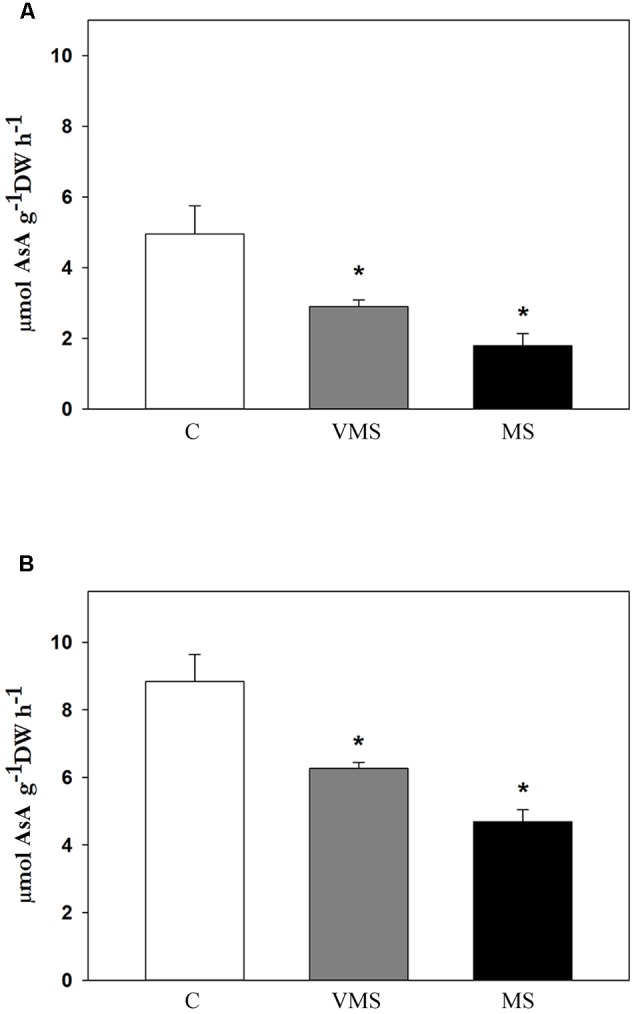
*In vivo* AsA biosynthesis rates in leaves of soybean plants under control (C), very mild (VMS) and mild (MS) stress conditions. Activity estimated as the difference in AsA content between at time 3 h vs. time 0 in untreated samples **(A)** and samples supplemented with GalL **(B)**. Values represent the average ± SE (*n* = 3 biological replicates). An asterisk (^∗^) denotes significant differences (Student’s *t*-test, *p* ≤ 0.05) with respect to C plants.

To test whether the observed decline in the levels of AsA could be explained by a reduction of AsA biosynthesis, AsA synthesis was investigated under *in vivo* conditions (**Figure 3**). Again, activity was only detected in leaves and stems, but not in root tissue. Since activity in leaves and stems showed a similar pattern, only leaf AsA synthesis is shown. AsA biosynthesis was negatively affected by drought, showing a progressive decline as Ψ_w_ values dropped (**Figure 3A**). To check whether there was a substrate limitation for GalLDH enzyme, activity was also measured adding the substrate of the reaction, GalL. In the presence of GalL, AsA biosynthesis increased in C plants around 78%, while drought stress led to a similar, progressive decline as the water deficit increased (**Figure 3B**). Although changes in cell wall permeability during drought cannot be discarded, results suggest that the observed reduction in AsA biosynthesis under water deficit is likely not related to a substrate limitation, opening the possibility of a regulation at the transcriptional or post-transcriptional level.

### Transcriptional Regulation of AsA Biosynthesis under Drought Stress

To gain further insights into the regulation of the biosynthesis of AsA at the transcriptional level, we analyzed the expression patterns of the genes involved in the pathway in soybean plants subjected to a progressive drought stress followed by a rewatering treatment (Song et al., 2016). Based on the Smirnoff-Wheeler pathway described for *A. thaliana*, we first searched for the putative orthologous genes in the soybean genome using a BLASTP (Basic Local Alignment Search Tool Protein^5^) approach. Since the first steps in the pathway generate compounds also involved in other metabolic pathways, we focused our analysis on the following enzymes: VTC1 (GDP-D-mannose pyrophosphorylase), GME (GDP-D-mannose 3′,5′-epimerase), VTC2/VTC5 (GDP-L-galactose phosphorylase), VTC4 (L-galactose-1-P phosphatase), GalDH (L-galactose dehydrogenase) and GalLDH. Unlike the situation in *A. thaliana*, the soybean genome contains multiple gene copies for each enzymatic step in the pathway, many of which are yet annotated as “unnamed protein product.” Four genes were found to code for VTC1 (Glyma02g41820, Glyma11g34550, Glyma14g07150 and Glyma18g03840), five genes for GME (Glyma01g09540, Glyma03g40720, Glyma10g30400, Glyma19g43410 and Glyma20g36740), the same set of four were retrieved when VTC2 and VTC5 were queried (Glyma02g46230, Glyma08g43200, Glyma14g02500 and Glyma18g10430), three for VTC4 (Glyma07g39620, Glyma09g01380 and Glyma15g12230), two for GalDH (Glyma07g30395 and Glyma08g06840) and two for GalLDH (Glyma02g27260 and Glyma10g17370). A known regulator of the pathway in *A. thaliana*, VTC3, for which only one candidate ortholog was found in the soybean genome, Glyma02g21970, was also included. A similar BLASTP analysis was carried out in the pasture legume *M. truncatula*, leading to the identification of two genes coding for VTC1 (Medtr3g069070 and Medtr5g080770), GME (Medtr1g080950 and Medtr7g115080), and VTC2/VTC5 (Medtr3g053020 and Medtr5g093390), and single-copy genes for VTC4 (Medtr2g026060), GalDH (Medtr4g092750) and GalLDH (Medtr1g050360). In the case of *AMR1* (At2g40860.1; Zhang et al., 2009), BLASTP searches did not retrieve any good match in either the soybean or the *M. truncatula* genome.

**FIGURE 4:**
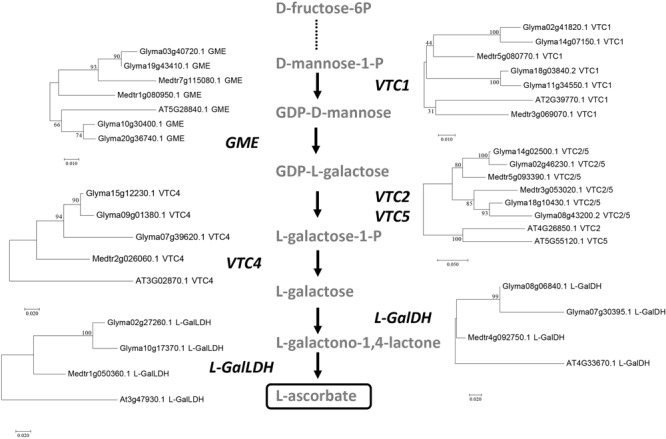
Phylogenetic analysis of the genes involved in the Smirnoff-Wheeler pathway from *A. thaliana, G. max*, and *M. truncatula*. Trees were constructed by aligning the predicted full-length amino acid sequences using MAFFT and subsequent phylogenetic analysis was carried out using MEGA7 by the maximum-likelihood method. Trees are drawn to scale, with branch lengths measured in the number of substitutions per site. Tree reliability was tested using 1000 bootstrap replicates. Bootstrap support values for each clade are indicated as percentages.

To provide further support to the identification of orthologous genes, we carried out a phylogenetic analysis of the predicted protein sequences for each step in the pathway from the two legumes and *A. thaliana* using the maximum likelihood method (**Figure 4**). The phylogenetic analysis clearly distinguishes the legume from the *A. thaliana* proteins and supports the likely gene endoduplication occurred in soybean, but not in *M. truncatula*, for genes encoding all the enzymes in the pathway.

**FIGURE 5:**
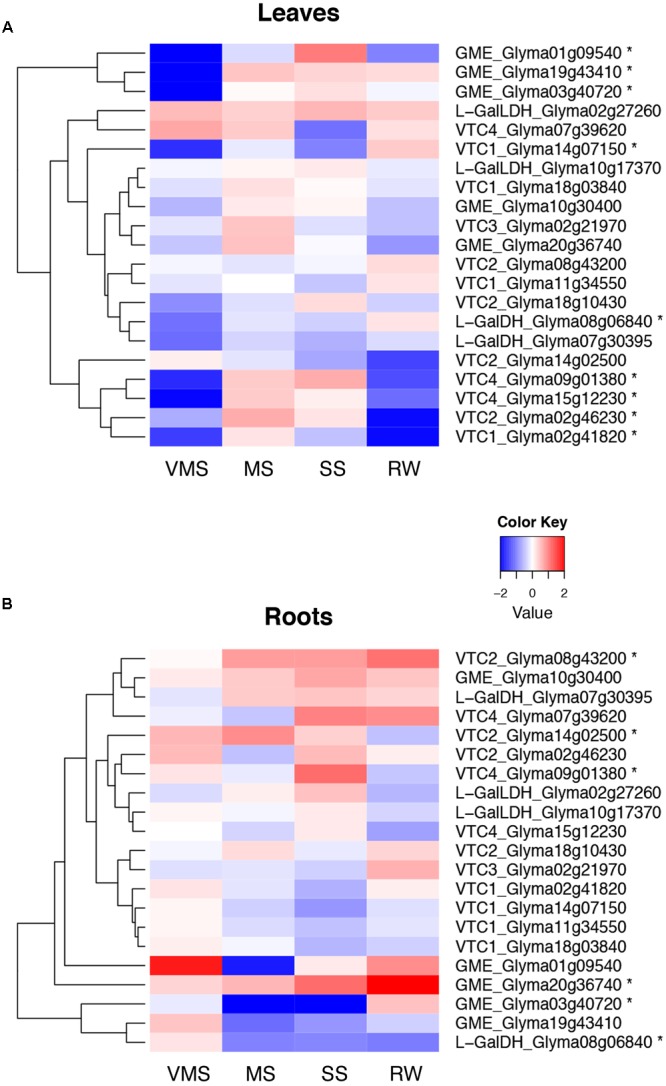
Expression of genes in the Smirnoff-Wheeler pathway in drought-stressed soybean plants. Hierarchical cluster representing the RNA-seq expression patterns of genes in the AsA biosynthetic pathway in leaf **(A)** and root tissue **(B)** under very mild (VMS), mild (MS), severe (SS) drought stress, and a subsequent recovery treatment (RW). An asterisk (^∗^) denotes significant differences between treatments and control plants (Student’s *t*-test, *p* < 5^∗^10^-5^).

Interestingly, the complete set of genes was found expressed in shoot and in root tissue both in soybean and *M. truncatula* (**Figure 5** and Supplementary Figure 1, respectively).

In terms of drought responses in soybean, the mildest stress treatment showed one of the strongest responses in leaf tissue, with a reduction in the transcript levels of most of the genes in the pathway compared to control plants. The expression of three out of the five *GME* genes and two *VTC4* genes, however, increased at later drought stages, a situation that was reverted upon rewatering of the plants. In contrast, the *GalLDH* genes, coding for the last enzyme in the pathway and supposedly one of the key regulators of the pathway, did not show a differential expression during drought (**Figure 5A**). In root tissue, however, the first stage of drought stress caused an increase in the transcript levels of most genes in the pathway, with the exception of the *GME* gene Glyma03g40720 and the *GalDH* gene Glyma08g06840, which showed a reduction in gene expression. Interestingly, the *VTC4* gene Glyma09g01380 showed an induction under severe drought conditions, a similar expression pattern to this observed in leaf tissue (**Figure 5B**). Regarding *VTC3*, only in root tissue there was a drought-related response of this putative regulator, whose transcript levels were lower under water deficit, a situation that was reverted by rewatering the plants.

To investigate whether this regulation at the transcriptional level was also found in other legume species, we queried the expression of genes in the Smirnoff-Wheeler pathway in the Medicago Gene Atlas database^6^ (Benedito et al., 2008), which has been now extended with expression data corresponding to a progressive drought stress followed by a recovery treatment (Zhang et al., 2014). In terms of drought responses, *M. truncatula* showed a differential response compared to soybean (Supplementary Figure 1). For instance, in leaves progressive drought caused a gradual increase in the expression of one of the *GME* genes, Medtr7g115080, while the levels *VTC1* gene Medtr5g080770 and the regulatory gene *VTC3* (Medtr1g050520) were reduced compared to well-watered plants. Interestingly, in roots drought induced the expression of the same *GME* gene observed in leaves, along with the *VTC2* gene Medtr5g093390, while there was a reduction of the transcript levels of the two *VTC1* genes (Supplementary Figure 1). It is worth noting that the two genes putatively coding for GME show an opposite pattern of expression both in roots and in leaves, which suggests a functional specialization of the genes.

## Discussion

In the current work, we analyzed the effects of drought stress in the biosynthesis of AsA in a crop of great economic relevance like soybean. The steady-state levels of AsA in a certain tissue are regulated via biosynthesis, degradation, recycling and transport of this antioxidant. Here we have measured the effects of drought stress in the regulation of biosynthesis both at the activity and transcriptional level, analyzing not only shoots, generally considered the main site of AsA biosynthesis in plants, but also root tissue. Knowledge on the AsA biosynthetic capacity of this underground organ is currently limited, despite being a key organ in terms of sensing a water deficit situation and triggering a response to drought stress. Through imposing a gradual water deficit, accompanied with a detailed plant physiological characterization (**Figure 1**), we observed a reduction of the levels of AsA both in leaves (**Figure 2A**) and stems (**Figure 2B**) of soybean plants. Such drought-induced decline in the levels of AsA has been also observed in the leaves of wheat (Bartoli et al., 1999) and several *Labiatae* species (Munné-Bosch and Alegre, 2003), as well as in symbiotic root nodules (Marino et al., 2007; Naya et al., 2007; Zabalza et al., 2008), but not in pea leaves (Moran et al., 1994). This lower content of AsA, however, was not correlated to increased levels of DHA, suggesting that it may be related to a reduced biosynthesis or increased transport of AsA. Indeed, results on *in vivo* GalLDH activity showed a significant and progressive reduction in the rates of AsA biosynthesis as lower Ψ_w_ values were reached (**Figure 3**). Thus, although alternative routes cannot be ruled out, we found a correlation between the levels of AsA and GalLDH biosynthetic rates in leaves of drought-stressed soybean plants.

To check whether there was a regulation of the pathway at the transcriptional level, we analyzed the expression patterns of enzymes in the Smirnoff-Wheeler pathway, including putative orthologs described as regulators in other plant species, in soybean and *M. truncatula* plants exposed to drought stress. Since the pathway has not been specifically described in legumes, we used the protein sequences of enzymes described in *A. thaliana* to identify the closest orthologous genes. Interestingly, we identified several closely related genes encoding for each of the enzymes in the pathway in soybean, as confirmed by the phylogenetic analysis (**Figure 4**). This genetic redundancy can be explained by the fact that the soybean genome has undergone at least two polyploidy events, estimated to occur ∼13 and ∼59 million years ago, which has led to 75% of the genes in the soybean genome being present in multiple copies (Roulin et al., 2013). It is interesting to note, however, how these multiple gene copies show differential patterns of expression, as observed for the soybean *GME* and *VTC4* orthologs (**Figure 5**), suggesting certain level of specialization at the functional level. These contrasting expression patterns may be also explained by a differential localization of the gene expression at the tissue level, a dimension that is lost when harvesting the complete tissue. Interestingly, the expression of genes coding for the full AsA biosynthetic pathway was detected not only in leaves but also in roots of both legume species. This suggests that although the vast majority of works in the literature have focused on AsA biosynthesis in leaves, roots have the potential of synthesizing this antioxidant compounds as well (Hancock et al., 2003; Liso et al., 2004), as shown in pea roots (Zabalza et al., 2007), *Lotus japonicus* root nodules (Matamoros et al., 2006), and a number of non-legume species.

The biosynthesis of AsA in plants is postulated to be regulated by several enzymes in the pathway, namely, GalLDH (Yabuta et al., 2007), GDP-D-mannose pyrophosphorylase (VTC1; Conklin et al., 1997, 1999; Veljovic-Jovanovic et al., 2001; Badejo et al., 2007, 2008; Wang et al., 2013), GDP-D-mannose 3′, 5′-epimerase (GME) in association with GDP-L-galactose phosphorylase (VTC2/VTC5; Dowdle et al., 2007; Wolucka and Van Montagu, 2007; Linster and Clarke, 2008; Bulley et al., 2009), the predicted F-box protein AMR1 (Zhang et al., 2009) and the protein kinase::protein phosphatase VTC3 (Conklin et al., 2013). These works are mainly based on the analysis of *A. thaliana* mutants defective in the production of AsA, or on correlations between the levels of AsA and gene expression of these enzymes in other plant species. Nevertheless, the regulation of the pathway under abiotic stress, particularly drought, has been barely studied. In the case of legume plants, this is the first work to our knowledge in which the genes coding for enzymes in the Smirnoff-Wheeler have been identified.

In the present study, *GalLDH* was not found differentially expressed in either soybean or *M. truncatula* plants under drought stress (**Figure 4B** and Supplementary Figure 1, respectively). If *GalLDH* is not regulated at the transcriptional level under water deficit conditions, it could be hypothesized that the observed decline in AsA levels and biosynthetic activity may be due to post-translational regulatory mechanisms of the GalLDH enzyme. This is further supported by the fact that *in silico* prediction of phosphorylation sites^7^ (Gao et al., 2010) identify two conserved Ser residues (Ser472 and 476, and Ser469 and 473 in Glyma.02G166300 and Glyma.10G104100, respectively; > %99 specificity) as potential targets for phosphorylation. Furthermore, several potential ubiquitylation sites are also found in the predicted soybean proteins [support vector machine probability > 0.83^8^ (Chen et al., 2013)]. Thus, one hypothesis to explain the reduction in the content of AsA and biosynthesis activity detected in leaves of drought-stressed soybeans could be differential phosphorylation of the enzyme or activation of its degradation via the ubiquitin-proteasome pathway.

VTC1 is encoded by a single gene in *A. thaliana*, while the soybean genome contains four putative orthologs and the *M. truncatula* genome two. Interestingly, there was a significant reduction in the level of transcripts in leaves of drought-stressed soybean plants for Glyma02g41820 and Glyma14g07150 (**Figure 5**), which correlated well with the observed decline in the content of AsA and biosynthesis in leaves. These genes are the closest orthologs to Medtr5g080770 in *M. truncatula*, whose expression was significantly and progressively reduced during the water deficit, together with the other gene coding for VTC1 in Medicago, Medtr3g069070 (Supplementary Figure 1).

Regarding the rest of genes tested, we could not find a direct link between their expression pattern and the reduction of AsA levels and biosynthetic rates measured under water-deficit conditions. In some cases transcript levels declined at VMS conditions but increased afterward. This is for instance the case of three out of the five genes encoding GME, the *VTC2/VTC5* Glyma02g46230 and two *VTC4* paralogs in soybean leaves. Furthermore, we often observed that putative paralogs presented differential gene expression patterns, suggesting the occurrence of functional specialization. For example, the expression of the *M. truncatula GME* gene Medtr7g115080 was induced both in leaves and roots, while the transcript levels of the other putative paralog, Medtr1g080950, were reduced in roots of drought stressed plants (Supplementary Figure 1B). Similarly, the transcript levels of *VTC3*, another known regulator of the pathway (Conklin et al., 2013), were found to decline only at early drought stages in soybean roots, but not subsequently (**Figure 5B**), while the content of AsA showed a continuous progressive decline as drought stress levels increased.

In summary, results presented here show a complex transcriptional regulation of the Smirnoff-Wheeler pathway in legumes under water-deficit conditions. Based on their expression patterns and correlation with the AsA levels and biosynthetic activity, our data suggest that GalLDH, together with VTC1, are the most likely candidates to play a role in the regulation of AsA biosynthesis in legume plants. Future work may provide answers to questions such as the role of post-transcriptional regulatory mechanisms in the drought-induced decline in GalLDH activity using targeted proteomic approaches (Wienkoop et al., 2008) or the individual contribution of the various paralogous genes. This knowledge will contribute to further understand the regulation of the pathway under water-deficit conditions to, ultimately, improve abiotic stress tolerance in legume crops.

## Author Contributions

AS, EL, and EG designed the experiments performed by AS, AZ, and EL. LS and HN provided RNA-seq expression data and discussed results. AS, EL, and EG discussed results and wrote the manuscript. All authors read and approved the final manuscript.

## Conflict of Interest Statement

The authors declare that the research was conducted in the absence of any commercial or financial relationships that could be construed as a potential conflict of interest.

## Footnotes

1 http://soybase.org/GlycineBlastPages/blast_descriptions.php

2 http://www.medicagohapmap.org/tools/blastform

3 http://mafft.cbrc.jp/alignment/server

4 http://mtgea.noble.org

5 http://blast.ncbi.nlm.nih.gov

6 http://mtgea.noble.org

7 http://musite.net

8 http://bioinfo.ncu.edu.cn/ubiprober.aspx

## Abbreviations

Ψ_w_: water potential
AsA: ascorbic acid
C: control
DHA: dehydroascorbate
DW: dry weight
FW: fresh weight
GalL: L-galactono-1,4-lactone
GalLDH: L-galactono-1,4-lactone dehydrogenase
MS: mild stress
VMS: very mild stress
WC: water content
