# Supplementary figures and images for: Drought Stress Causes a Reduction in the Biosynthesis of Ascorbic Acid in Soybean Plants
Source: Front Plant Sci. 2017 Jun 15;8:1042. doi: 10.3389/fpls.2017.01042 (PMC5471321; doi:10.3389/fpls.2017.01042)

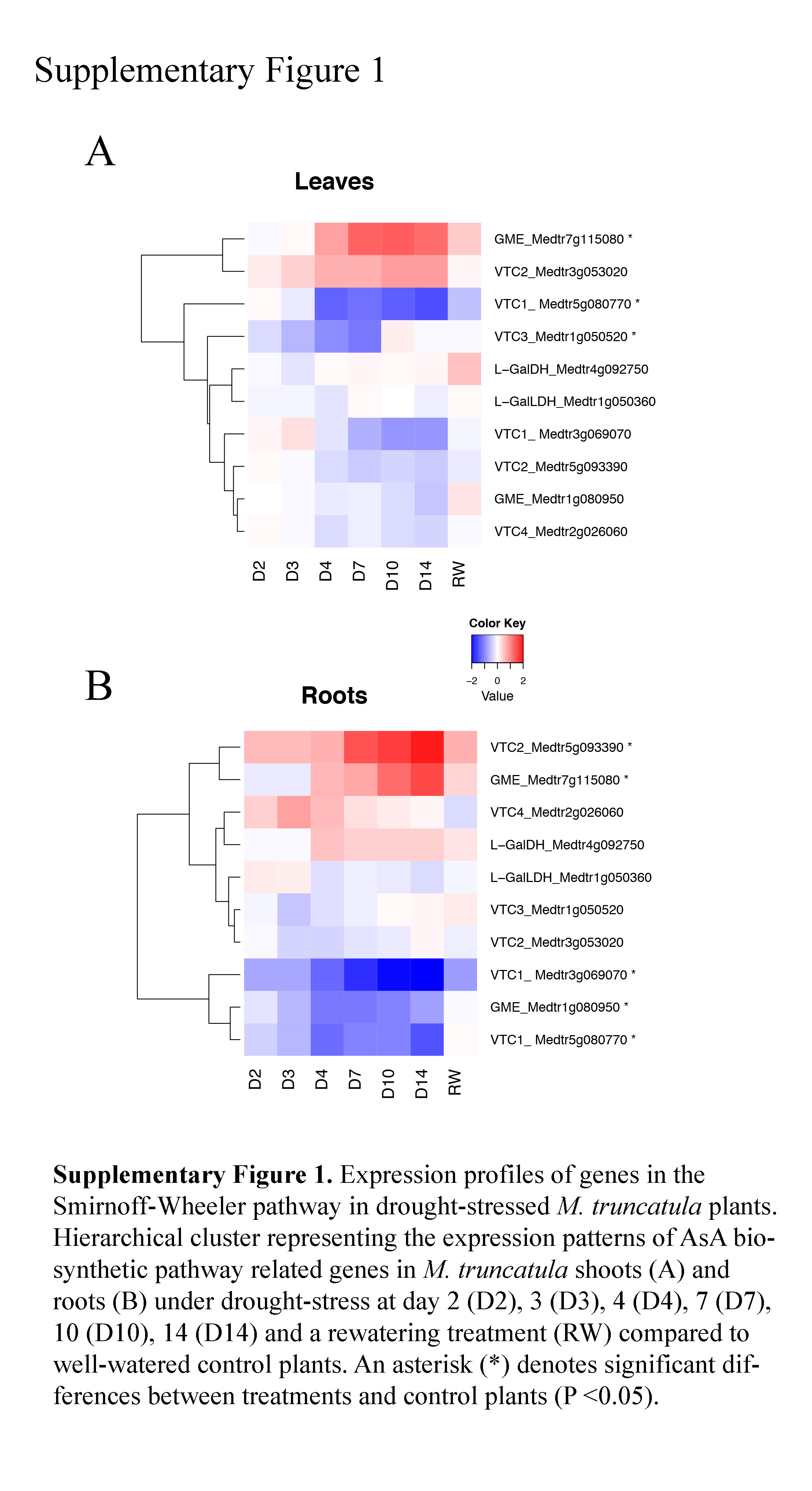

Supplement: Supplementary file 1 [file Image_1.JPEG]
